# 趋化素样因子超家族成员在肿瘤中的研究进展

**DOI:** 10.3779/j.issn.1009-3419.2023.106.01

**Published:** 2023-01-20

**Authors:** Gang XIE, Jing CHENG, Junping ZHANG

**Affiliations:** 030032 太原，山西医科大学第三医院（山西白求恩医院，山西医学科学院，同济山西医院）; Third Hospital of Shanxi Medical University, Shanxi Bethune Hospital, Shanxi Academy of Medical Sciences, Tongji Shanxi Hospital, Taiyuan 030032, China

**Keywords:** 趋化素样因子, CKLF, CMTM, 肿瘤, 生物标志物, Chemokine-like factor, CKLF, CMTM, Tumor, Biomarker

## Abstract

趋化素样因子超家族成员（chemokine-like factor-like MARVEL transmembrane domain containing member/chemokine-like factor superfamily member, CMTM/CKLFSF）是连接趋化因子和跨膜超家族的一组新的蛋白家族，包括CKLF和CMTM1-CMTM8。CMTM除了具有广泛的趋化活性外，还与造血系统、免疫系统以及肿瘤的发生、发展和转移密切相关。CMTM蛋白参与癌症发展中的关键生物学过程，包括生长因子受体的激活和循环、细胞的增殖与转移以及肿瘤免疫微环境的调节。随着研究发现CMTM4、CMTM6可作为程序性死亡配体1（programmed cell death ligand 1, PD-L1）的调控器，关于CMTM与肿瘤之间的关系重新成为研究热点。本文对CMTM家族成员在癌症中作用进行综述，特别是在肿瘤生长、转移和免疫逃逸中的研究进展，并总结CMTM与非小细胞肺癌之间关系的最新研究结果，探讨CMTM作为新型药物靶点或生物标志物的潜在临床价值。

## Abstract (en)

Chemokine-like factor-like MARVEL transmembrane domain containing member/chemokine-like factor superfamily member (CMTM/CKLFSF) including CKLF and CMTM1-CMTM8 are a new family of proteins linking chemokines and transmembrane superfamilies. CMTM not only have broad chemotactic activities, but also associate with hematopoietic system, immune system, and tumor development and metastasis closely. CMTM proteins are involved in key biological processes of cancer development, which include activation and recycling of growth factor receptors, cell proliferation and metastasis, and regulation of the tumor immune microenvironment. This is a new focus of research on the relationship between CMTM and tumors, because CMTM4/CMTM6 can be considered as a regulator for programmed cell death ligand 1 (PD-L1). This paper reviews the role of CMTM family members on cancer, especially in tumor growth, metastasis and immune escape, summarize the latest findings on the relationship between CMTM and non-small cell lung cancer, and explores the potential clinical value of CMTM as a novel drug target or biomarker.

趋化素样因子超家族成员（chemokine-like factor-like MARVEL transmembrane domain containing member/chemokine-like factor superfamily member, CMTM/CKLFSF）由北京大学人类疾病基因研究中心发现^[1]^，包括9个基因：CKLF和CMTM1-CMTM8（即CKLFSF1-CKLFSF8）。CKLF由四个外显子组成，位于染色体16q22上，维持四种可供选择的RNA剪接形式，因此又分为CKLF1-CKLF4。MARVEL（MAL and related proteins for vesicle trafficking and membrane link）是一种新鉴定的四次跨膜结构域^[2]^，而CMTM则是以MARVEL结构域为特征的具有趋化活性的新型蛋白，在整个免疫系统中广泛表达。起初认为CMTM家族成员与自身免疫性疾病有关，随后发现在男性生殖系统和心血管系统中CMTM也发挥着重要的生物学作用^[3,4]^。后来，诸多研究^[5,6]^表明肿瘤和正常组织的CMTM存在差异表达，且与肿瘤分级、分期、转移和总生存期有关，对肿瘤的预后有一定的预测价值，但CMTM对肿瘤的作用并未引起人们的重视。

随着Nature杂志报道^[7,8]^CMTM4/CMTM6可降低程序性死亡配体1（programmed cell death ligand 1, PD-L1）的泛素化，增加PD-L1蛋白的半衰期，增强PD-L1^+^肿瘤细胞抑制T细胞的能力，CMTM4/CMTM6可作为PD-L1的调控器，CMTM与肿瘤之间的关系逐渐成为研究热点。进一步研究^[9]^发现，由于CMTM家族成员结构和功能的多样性以及肿瘤之间的异质性，不同的CMTM对多种恶性肿瘤的癌基因和肿瘤抑制因子的作用存在明显差异。但总的来说，CMTM可作为治疗人类癌症的潜在治疗靶点。本文旨在探索CMTM的结构和功能，重点综述CMTM在参与肿瘤免疫微环境、介导信号转导、调节肿瘤侵袭和转移等方面的研究进展，总结CMTM与非小细胞肺癌（non-small cell lung cancer, NSCLC）之间关系的最新研究结果，并探讨其作为生物标志物的临床价值以及潜在的预后价值。

## 1 结构特点与表达

大多数CMTM基因转录的RNA产物具有多种可变剪接形式，但所有翻译后的蛋白质都包含髓鞘和淋巴细胞蛋白（myelin and lymphocyte protein, MAL）和MARVEL结构域^[1]^。与经典四次跨膜蛋白相比，MARVEL结构域的两个细胞外襻都较小，第三跨膜区起始部位的保守酸性残基对其功能有重要影响，MARVEL结构域参与囊泡运输、膜连接等多种生物学过程，其蛋白表达或功能异常可导致多种疾病的发生发展^[2]^。CKLF和CMTM1-CMTM8基因位于：人3号（CMTM6、CMTM7、CMTM8）、14号（CMTM5）和16号（CMTM1、CMTM2、CMTM3、CMTM4）染色体和小鼠8号、9号和14号染色体^[1,10]^。CMTM1-CMTM8编码的蛋白质与经典趋化因子和四跨膜蛋白超家族（transmembrane 4 superfamily, TM4SF）的结构相似。CMTM1包含一个C-C基序，与其他CMTM相比，CMTM1和趋化因子具有更高的序列同一性，而CMTM8与趋化因子的序列同一性最低，CMTM2-CMTM7介于两者之间^[1]^；但是，CMTM8与TM4SF的氨基酸相似性为39.3%。

CMTM1-CMTM4在睾丸、骨髓和外周血细胞的中高度表达，尤其是静息CD19^+^细胞和活化的外周血单核细胞。CMTM3、CMTM5、CMTM7、CMTM8在正常成人和胎儿组织中可广泛表达^[6,11]^，但在大多数癌细胞系中，其表达随着启动子区域DNA的甲基化逐渐降低^[12]^。有研究^[9]^发现除CMTM3外，7种CMTM家族成员在NSCLC中有差异表达并与其恶性进展密切相关。

## 2 CMTM与肿瘤免疫微环境

### 2.1 CMTM与免疫细胞

既往研究^[13]^表明，CKLF1可以通过MAPK途径激活中性粒细胞，当加用抗-CKLF1抗体时，中性粒细胞的数量和活性随之减少。C19和C27是CKLF1的两个多肽，用C19和C27刺激未成熟树突状细胞（immature dendritic cell, ImDC），参与ImDC的抗原提呈，T细胞增殖和γ干扰素（interferon-gamma, IFN-γ）的产生也会受到影响。C19和C27也可作用于成熟DC细胞，上调HLA-DR的表达，促进白介素12（interleukin 12, IL-12）的分泌，而对CD80、CD83和CD86无明显影响。在研究活化T淋巴细胞中CKLF1的表达谱时，Li等^[14]^证明CKLF1在活化的CD4^+^和CD8^+^细胞中上调，而在CD19^+^细胞中没有明显变化。

CMTM3和CMTM7高表达于免疫器官及造血系统，在B细胞上高表达，是B细胞连接蛋白（B cell linker protein, BLNK）的结合伙伴，与B细胞受体（B cell receptor, BCR）结合，参与BLNK介导的B淋巴细胞信号转导，CMTM3可与DC2.4细胞中的SLP76蛋白结合，而CMTM7缺失导致B-1a细胞在过渡阶段发育停滞^[15]^。

CMTM可能通过作用于免疫细胞和免疫分子，在抗磷脂综合征（antiphospholipid syndrome, APS）的发生发展过程中发挥潜在作用。研究^[16]^发现，APS患者中性粒细胞的CMTM2和CMTM6表达上调。此外，CKLF1可能通过与C-C趋化因子受体4相互作用而参与关节炎的发生与发展^[17]^。

研究^[18]^表明CMTM可通过影响免疫细胞，如DCs、B细胞、共同淋巴样祖细胞（common lymphoid progenitors, CLPs）、巨噬细胞、肥大细胞、Th2细胞和单核细胞的浸润，改变肿瘤免疫微环境，对II级/III级脑胶质瘤患者的预后产生一定影响。中山大学肿瘤中心发现高CMTM6水平与适应性免疫表型的肿瘤微环境的激活、活化CD4^+^、CD8^+^ T细胞的浸润、结直肠癌患者总生存率（overall survival, OS）和无进展生存期（progression-free survival, PFS）的延长有关^[19]^。CMTM可与多种免疫细胞相互作用，主要表现为参与DC细胞的抗原递呈、激活中性粒细胞和B淋巴细胞的转导信号、影响免疫细胞的浸润。

### 2.2 CMTM4/CMTM6调控PD-L1

PD-L1高表达于肿瘤细胞，通过与T细胞膜上的程序性死亡受体1（programmed cell death 1, PD-1）相互作用，可有效抑制抗原特异性CD8^+^ T细胞的功能，帮助肿瘤细胞逃避机体免疫系统的识别和攻击。美国食品药品监督管理局（Food and Drug Administraton, FDA）批准多种针对PD-L1、PD-1的抗体上市，在肿瘤免疫治疗中取得了巨大成功，目前，阻断PD-L1、PD-1的相互作用是肿瘤免疫治疗的热点。尽管PD-L1在肿瘤免疫微环境中癌细胞的免疫逃逸过程中起着关键作用，但我们对PD-L1蛋白调控的理解是有限的。

CMTM6是一种普遍表达的蛋白，与PD-L1结合并维持其细胞表面表达，在肿瘤微环境中有重要作用。通过单倍体遗传筛选的3型跨膜蛋白CMTM6作为PD-L1蛋白的调节因子，可通过IFN-γ信号通路有效影响PD-L1的表达，CMTM6的表达下调会导致人肿瘤细胞和原代人树突状细胞中PD-L1蛋白的表达受损。值得注意的是，CMTM6可增加PD-L1蛋白含量，对PD-L1表现出明显的特异性，但不影响PD-L1的转录水平，不影响细胞表面主要组织相容性复合体（major histocompatibility complex, MHC）I类递呈抗原。抑制CMTM6表达能够下调PD-L1、显著降低肿瘤细胞对T细胞活性的抑制能力，进一步研究^[7]^表明细胞表面的CMTM6与PD-L1蛋白结合，还可减少其泛素化，延长PD-L1蛋白的半衰期，在质膜上的CMTM6与PD-L1结合，保护PD-L1在细胞内体循环中免受溶酶体降解。

CMTM4是CMTM家族的另一个成员，与CMTM6具有55%的同源性，被鉴定为PD-L1表达的正调节剂。通过对CMTM6基因缺失细胞的单倍体遗传修饰筛选和遗传互补实验发现CMTM4和CMTM6一样，具有调节PD-L1的功能，而其他被测试的CMTM成员目前则不具有该功能。与CMTM6在调节PD-L1蛋白的作用一致，CMTM4增强了PD-L1^+^肿瘤细胞抑制T细胞的能力^[8]^。

结肠癌组织中CMTM6的表达明显高于正常结肠组织，CMTM6的高表达与较低的病理分期和较多的CD4^+^/CD8^+^肿瘤浸润淋巴细胞有关，PD-L1在结肠癌组织中呈低水平表达，而在肿瘤间质中高表达，CMTM6和肿瘤间质中PD-L1的共表达状态将结肠癌患者分为低、中、高风险组，其中高表达组的患者生存时间最长，CMTM6/PD-L1在辅助化疗组中比未化疗组有着更好的预后价值^[19]^。在胃癌的研究^[20]^中，通过免疫组织化学检查105例根治性手术切除的II期/III期胃癌患者CMTM6的表达，发现CMTM6高表达组的OS明显低于CMTM6低表达组，并且CMTM6表达与PD-L1表达呈正相关。在肝细胞癌的研究^[21]^中发现CMTM4缺失的HCC肿瘤患者对抗PD-L1治疗更敏感，这提示CMTM4表达水平可作为患者抗PD-L1治疗反应的预测指标之一，干扰CMTM4表达可能有助于提高肝癌患者抗PD-L1免疫治疗的临床效益。同样，CMTM4敲低则可抑制IFN-γ诱导的头颈部鳞状细胞癌中PD-L1的表达，CMTM4表达与基质中CD8和PD-L1细胞密度呈正相关，研究^[22]^表明CMTM4可能在调节癌症干细胞样表型以及PD-L1的表达中起重要作用。这些研究数据表明PD-L1依赖CMTM4、CMTM6有效地发挥其抑制功能，CMTM4、CMTM6可作为潜在的免疫治疗新靶点和预测预后的生物学标志物。PD-L1单抗（如阿替利珠单抗、度伐利尤单抗等）目前正广泛应用于治疗局部晚期或转移性NSCLC，而CMTM4、CMTM6作为PD-L1的调节剂，其在提高PD-L1单抗治疗NSCLC患者的长期有效率及克服PD-L1单抗的耐药性方面需进一步深入探究。

## 3 CMTM介导的信号传导

### 3.1 CMTM调节表皮生长因子受体（epidermal growth factor receptor, EGFR）

EGFR信号传导调节上皮组织的发育和稳态，被认为是肿瘤抗药性的生物标志物。当EGF作用于EGFR时，EGFR被磷酸化，从而导致控制细胞增殖、分化和迁移的细胞内级联信号的激活。EGFR信号起始于质膜，经内化、泛素化和随后的降解，参与负调控调节^[23]^。CMTM3、CMTM5、CMTM7在肿瘤中被认为是肿瘤抑制因子，通过调节EGFR信号在肿瘤微环境中发挥抑制作用。CMTM7通过参与调控EGFR的内吞作用，抑制EGFR再循环，从而降低细胞表面EGFR水平，抑制下游PI3K/AKT信号通路，对ERK信号通路无影响；除此之外，研究^[11]^发现CMTM5和CMTM7具有协同作用，促进EGFR内化并抑制EGFR介导的PI3K/AKT信号通路。CMTM3激活Rab5，促进早期内体融合，下调细胞表面EGFR蛋白水平，从而调节EGFR内吞作用^[24]^。研究^[25]^报道，脊索瘤组织的CMTM3表达下调，CMTM3通过激活TP53信号通路和阻断EGFR/STAT3信号通路，抑制上皮间质转化（epithelial-mesenchymal transition, EMT）过程，从而抑制脊索瘤的发生和发展。EGFR突变是晚期NSCLC常见的突变类型，也是NSCLC靶向治疗的重要靶点，CMTM3、CMTM5、CMTM7通过调节EGFR信号而成为NSCLC治疗领域的潜在靶点。

### 3.2 CMTM影响细胞周期

细胞周期包括G_1_期、S期、G_2_期、M期及G_0_期，有一个称为细胞周期控制系统的复杂信号网络精细调节，参与调控信号的主要成分是细胞周期蛋白和细胞周期蛋白依赖性激酶（cyclin-dependent kinase, CDK）。细胞周期失调可导致肿瘤中的异位细胞增殖。因此，细胞周期蛋白和CDK已成为肿瘤的治疗靶点。CMTM4的过表达使G_2_期/M期细胞周期停滞和p21上调，显著抑制肾细胞癌（renal cell carcinoma, RCC）细胞系786-O细胞的生长，并抑制细胞迁移^[26]^。在RCC中CMTM5表达下调，通过恢复CMTM5表达可诱导G_0_期-G_1_期细胞周期阻滞的发生，从而抑制RCC细胞的增殖^[27]^。体外研究^[28]^表明，CMTM7的过表达抑制肝癌细胞的生长和迁移。进一步分析显示CMTM7抑制AKT信号传导并诱导肝癌细胞G_0_期/G_1_期细胞周期停滞，可能是由于p27表达增加，细胞周期蛋白D1、CDK4和CDK6水平降低所致。CMTM7通过抑制细胞周期进程在肝癌中发挥肿瘤抑制剂的作用^[28]^。总的来说，CMTM4、CMTM5、CMTM7可使细胞停滞在不同周期，影响细胞生长和迁移。

## 4 CMTM参与EMT

EMT是一个上皮细胞失去其上皮特征，连接并重组其细胞骨架的发育过程。EMT增加细胞流动性，使癌细胞获得侵袭性，这在耐药性和肿瘤转移中发挥重要作用。CMTM4可能在调节EMT、肿瘤干细胞表型和PD-L1表达中发挥重要作用，且EMT过程与PD-L1表达相关，敲除CMTM4可使EMT过程延缓，抑制肿瘤细胞的迁移和侵袭能力^[22]^。CMTM3通过EGFR/STAT3调控EMT从而抑制脊索瘤的发生^[25]^。在肝癌的研究^[29]^中阻断CMTM2的表达可显著促进HUH-7和SMMC7721细胞增殖，其癌细胞转移与CMTM2下调有关，CMTM2的低表达诱导EMT过程，增强肝癌细胞的侵袭和迁移能力。研究^[30]^发现CMTM3、CMTM6、CMTM7的表达与乳腺癌细胞系的EMT的相关评分呈正相关，EMT可通过诱导CMTM6和CMTM7调节乳腺癌表面PD-L1表达。总之，CMTM可通过影响EMT过程来调节肿瘤的侵袭和转移。

## 5 CMTM与肿瘤预后

CMTM家族在评估病理分期、确定治疗策略方面具有相当大的临床价值，可作为预测NSCLC、乳腺癌、肝癌和胃癌等癌症预后的生物学标志物。CMTM4在肝细胞癌、肺腺癌组织中表达下调，是影响肝细胞癌、肺腺癌患者OS的独立预后因素，CMTM4阴性表达的患者的生存期短于CMTM4阳性表达的患者^[31,32]^。CKLF是CMTM家族中第一个被发现的成员，也是人类肿瘤中一个具有很大希望的治疗靶点^[33]^。通过整理分析ONCOMINE、GEPIA、Kaplan-Meier Plotter和cBioPortal数据库中乳腺癌患者CMTM的转录和生存数据，表明CMTM5、CMTM7可以作为预测乳腺癌预后的生物标志物和潜在的治疗靶点^[34,35]^。CMTM的mRNA表达与胃癌患者的临床参数（包括肿瘤分期、淋巴结转移状况、幽门螺杆菌感染状况和肿瘤分级）密切相关，CMTM3/CMTM5 mRNA水平升高与较差的总体生存率、无进展生存率和进展后生存率显著相关，提示CMTM的表达模式可能是一种新的影响胃癌患者预后的因素^[36]^。研究^[37]^利用免疫组化证实CMTM6、CMTM8在卵巢癌组织中高表达，CMTM6高表达与PD-L1密切相关，CMTM8高表达与Ki-67密切相关，并可显著降低总生存率和无进展生存率，CMTM1、CMTM2、CMTM3、CMTM5、CMTM8可作为判断卵巢癌预后的生物标志物。总之，CMTM作为预测患者预后和治疗效果的新的生物标志物，在肿瘤方面具有潜在的研究价值。

## 6 CMTM与NSCLC

Si等^[38]^发现在NSCLC中，亚型CMTM1_V17的高表达会降低患者对新型辅助化疗的药物敏感性，同时对患者术后生存率造成严重影响。Li等^[39]^通过免疫组织化学染色的方法证明，与配对的相邻正常肺组织相比，CMTM2在肺腺癌组织中的表达明显减少，并在小鼠模型中证实CMTM2对肿瘤生长的抑制作用。在研究肺癌的模型中，Tian等^[40]^发现CMTM4表达可显著影响肺鳞癌和腺癌患者的OS。而在人蛋白质数据库中发现CMTM5表达减少与肺腺癌和鳞状细胞癌的较低存活率相关^[9]^。Zugazagoitia等^[41]^研究表明CMTM6和PD-L1的高共表达水平与NSCLC预后较好相关。Koh等^[42]^研究证明，高CMTM6蛋白表达的NSCLC患者比低CMTM6蛋白表达的患者具有更好的OS，CMTM6升高可预测NSCLC患者对PD-1抑制剂的临床反应。并且，相关研究^[43]^在mRNA和蛋白两个水平验证了CMTM6与PD-L1存在正相关关系，下调的CMTM6导致肺腺癌细胞发生突变。CMTM7是NSCLC生存的独立预后因素，进一步研究^[44]^发现CMTM7基因缺失导致Rab5活性降低，导致肿瘤细胞增殖。有研究^[9,45]^报道CMTM8经常在肺癌中下调或缺失，其表达下调与肺鳞癌生存率降低有关。而CMTM3是否参与调控NSCLC分子机制仍需进一步研究。CMTM4、CMTM6作为抑癌基因对NSCLC患者的预后生存有明显的影响，可能与PD-1/PD-L1途径有关，但更深入的机制还有待探索。

## 7 总结与展望

CMTM在许多生物过程中发挥关键作用，比如维持肿瘤免疫微环境，调节PD-L1蛋白、EGFR信号通路、增殖信号传导以及参与侵袭和转移。CMTM在人类肿瘤和正常组织中有不同的表达谱，不同的CMTM成员在肿瘤的发生与发展中作用不同，可发挥肿瘤的双向调节作用。CMTM在肿瘤发展和转移中的作用已被初步探究，但要明确调节CMTM表达的分子机制还需要进一步研究。很多实验数据表明CMTM在癌症发展中发挥了重要作用，但没有证据表明异常CMTM表达是否直接干扰化学治疗、放射治疗和免疫治疗的效果。我们对CMTM在肿瘤微环境中的作用的认识仍是冰山一角。临床上应用不同CMTM家族成员或其促进/抑制剂是否能有效控制肿瘤的发生发展或转移，如何将其应用于临床治疗中仍然是需要我们不断探索的关键问题。此外，有必要进一步研究CMTM对其他重要膜分子尤其是免疫激活性受体、免疫抑制性受体是否具有调控作用，研发CMTM4、CMTM6的阻断型抗体或小分子化合物可为肿瘤免疫治疗提供新的思路和方法。
